# Assessment of Patients' Views on Drug Benefits and Risks: An Interview Study with Cardiovascular Patients

**DOI:** 10.1155/2022/6585271

**Published:** 2022-11-14

**Authors:** Ines Wakob, Ina Wintsche, Annett Frisch, Yvonne Remane, Ulrich Laufs, Thilo Bertsche, Susanne Schiek

**Affiliations:** ^1^Clinical Pharmacy, Institute of Pharmacy, Medical Faculty, Leipzig University, Leipzig, Germany; ^2^Drug Safety Center, Leipzig University and Leipzig University Hospital, Leipzig, Germany; ^3^Pharmacy Department, Leipzig University Hospital, Leipzig, Germany; ^4^Klinik und Poliklinik für Kardiologie, Universitätsklinikum Leipzig, Leipzig, Germany

## Abstract

Better and balanced information strategies supporting cardiovascular patients' adherence are required. Cardiovascular drugs have outstanding morbidity and mortality benefits. This can be counteracted by patients' perceptions of risks. Drug information should help the patient but not fuel unwarranted fears. We performed a cross-sectional survey of patients admitted to a cardiology ward. We evaluated (i) the patients' general benefit-risk estimation of their pharmacotherapy; (ii) views on benefits; (iii) views on risks; and (iv) information sources. Additionally, we assessed aspects of anxiety and depression with the Patient Health Questionnaire-4 (PHQ-4). (i) 67 patients (66%) rated expected drug benefits higher than potential risks. (ii) 72% of benefits motivated the patients to take their medication as prescribed. Patients more frequently mentioned surrogate markers as benefits than clinical benefits (*p* < 0.001). (iii) 56% of risks mentioned were perceived as bothersome and 35% as concerning. Risks were more often perceived as bothersome and concerning by patients with higher PHQ-4 scores (*p*=0.016). (iv) Physicians were the most frequent information source of benefits (92% of patients) and risks (45%), and pharmacy staff for 27% and 14%, respectively. Laymen or media served as sources of information on benefits in 39%, for risks in 40%, and package leaflets in 26% and 36%. 42% of the patients would like to receive more information on benefits versus 27% on risks. Our results suggest that knowledge of benefits motivates patients to take their drugs as prescribed. There is already good information on surrogate markers for process control with active patient involvement. However, a lack of knowledge still exists in relation to clinical benefits. Regarding risks, it has been shown that patients with higher PHQ-4 scores are more likely to be bothered or concerned. Both emphases on clinical benefits and individualization depending on PHQ-4 scores may be valuable resources for patient counseling to support adherence.

## 1. Introduction

Due to their high prevalence and mortality, cardiovascular diseases are of great interest in industrialized countries [1]. Their corresponding pharmacotherapies often have outstanding effects on morbidity and mortality as they avoid myocardial infarctions and strokes. These effects are not immediately noticeable to patients. However, in order to achieve such positive effectiveness in the long term, they have to take their medication conscientiously over a long period of time. Therefore, it is not surprising that adherence decreases over time, which has been shown to especially affect patients with cardiovascular diseases [2]. In this regard, diverse forms of nonadherence occur, e.g., patients modifying their dosages or stopping their therapy altogether by themselves. While nonadherence represents a well-known issue [3], better strategies for more balanced drug information could, on the one hand, provide the patient with knowledge about benefits and risks and, on the other hand, help to prevent unjustified fears and support adherence.

Patients' attitudes toward drugs and, thereby, their adherence is influenced by information. The psychological mechanisms of nocebo and placebo need to be considered in this context [4–6]. For statin therapy, for example, it has been shown that fewer risks were reported in blinded than in open observational studies [7]. Studies have also shown that laymen's information can incite fears of risks [7, 8]. Such anxiety is likely to decrease adherence, as shown for statins [9, 10]. Due to the increasing access to digital information and patients' individual needs for information on drugs [11, 12], healthcare professionals face additional challenges in information and counseling.

Adherence is primarily predicted by patients' attitudes toward their medicines [13]. It is further influenced by two drug-related aspects. First, nonadherence is often caused by risk factors for events that have actually occurred or that the patients are afraid of [14–16], although they may never have experienced them themselves. Second, the patients should also be aware of the benefits of their pharmacotherapy. An imbalance between experienced or expected risks and benefits can negatively influence adherence [13, 17]. Strategies to support patients in their challenge to stay motivated should therefore include the patients' views on both drug risks and drug benefits. Furthermore, patients' individual characteristics were found to have an impact on adherence [4, 11, 18]. In this respect, depression and anxiety are of special interest as influencing factors [19, 20]. This is because depression represents frequent comorbidity in cardiovascular diseases and is associated with lower adherence and worse control of low cholesterol levels and blood pressure values [21, 22]. One very short form for routine screening of the risk for depression and anxiety is the Patient Health Questionnaire-4 (PHQ-4).

We, therefore, investigated the patients' views on cardiovascular drug benefits and risks. The relevant sources of information were reviewed and separated into professional and laymen sources. This way, this study should help to derive more tailored patient information strategies to support cardiovascular patients in their adherence.

## 2. Methods

### 2.1. Ethics Vote

The Ethics Committee of the Medical Faculty of Leipzig University (223/18-ek) approved the study protocol. This study conforms to the ethical standards of the 1975 Declaration of Helsinki. All patients participated voluntarily, and written informed consent was obtained from all patients prior to inclusion. In case the medication review or the interview indicated potential patient safety aspects concerning risks or nonadherence, the attending physician was informed immediately.

### 2.2. Setting

Our survey was performed from July to/until October 2018 in a cardiology department with 46 beds in a university hospital offering tertiary care. The department offers interventional cardiology and the diagnosis and treatment of cardiac insufficiency, coronary heart disease, arrhythmias, and all other cardiovascular diseases.

### 2.3. Participants

We consecutively enrolled all patients admitted to the cardiology department who were more than 18 years old and fully contractually capable. Further inclusion criteria were that patients took their medication on their own responsibility, had sufficient German language skills, and had been prescribed cardiovascular pharmacotherapy for at least four weeks. Exclusion criteria were patients' refusal to participate; cognitive impairment; communication barriers (e.g., blind and deaf); and the absence of one of the inclusion criteria. If the patient shared a room with another patient who had already participated in the study and an information exchange was expected, he or she would not be included.

### 2.4. Study Design

We performed a cross-sectional study by conducting semistructured face-to-face interviews with inpatients. The interview was supplemented by a subsequent written assessment of anxiety and depression using the PHQ-4 [23].

### 2.5. Development of the Interview Guide

An expert panel consisting of five pharmacists developed the interview guide. The interview guide was pretested incrementally with pharmacists (*n* = 6) and medical laymen (*n* = 11) not involved in study conception and conduction. Pretest techniques were used including sorting, paraphrasing, comprehension probing, category selection probing, and confidence rating. Due to the pretest, changes in vocabulary have been made in order to meet the patients' level of understanding. Therefore, benefits were referred to as “positive effects” and risks as “negative effects.” The pretest was additionally used to test the practicability of the semistructured interview. None of the participants taking part in the pretests were involved in the main survey. The results of the pretests were not included in the data analysis.

The interview included 4 main themes:

- Patients' general estimation of therapeutic benefits and risks.
- Patients' views on drug benefits and whether they motivate the patient to take a drug as prescribed. The patient could mention the benefits actively (without suggestion) or subsequently passively based on a predefined list of suggestions for common benefits of cardiovascular therapy.
- Patients' views on drug risks and whether they were perceived as bothersome, concerning, or causing the patient to take medication differently than prescribed. Bothersome and concerning risks were additionally assessed because these parameters could negatively influence adherence. The patients could mention the risk actively (without suggestion) or subsequently passively based on a predefined list of suggestions for common risks of cardiovascular therapy.
- Used information sources on cardiovascular therapy about benefits and risks. Additionally, we asked the patients whether they would like to get more information about the benefits and risks of their cardiovascular therapy. For details on the interview guide, see Supplementary Appendix 1.

Benefits and risks included both already experienced events and those the patients had heard or read about. Adherence was addressed indirectly by asking the patients whether the benefit motivates them to take their medication as prescribed or whether the risk caused the patients to take their medication differently than prescribed, respectively.

### 2.6. Data Collection

All patients who met the inclusion criteria were personally invited to participate. All interviews were performed by the same person (pretrained pharmacist) from July 20, 2018, to October 10, 2018. The interview was carried out face-to-face in a semistructured way to enable the patient to speak in an unbiased manner.

Before the study interview, the patient's medication was reviewed for cardiovascular medication using patient chart documentation. Additionally, the patient was asked about his or her current medication.

At the end of the interview, the patient was asked for sociodemographic data. Following the interview, the patient was invited to fill out the PHQ-4.

The interviewer documented the patient's answers in written form. To minimize recall bias, notes were reviewed immediately after the interview and answers were coded and entered into an electronic data sheet (based on Microsoft Office Excel 2016). For quality assurance, every digitalized interview was checked by a second person (pharmacist) from the study team in order to exclude transfer errors.

Drugs were classified based on the Anatomical Therapeutic Chemical (ATC) classification of the World Health Organization (WHO) [24]. We considered the following ATC groups as “cardiovascular”: A10, B01, C02, C03, C07, C08, C09, and C10. Benefits were classified based on the 10th revision of the International Classification of Diseases (ICD-10) by the WHO [25]. Risks were classified based on the Common Terminology Criteria for Adverse Events (CTCAE) classification (v5.0 Publish Date: November 27, 2017) [26].

### 2.7. Data Analysis

Benefits were separated into surrogate markers and clinical benefits. To assess differences between surrogate markers and clinical benefits, we first counted the patients with the respective drugs (e.g., patients with antihypertonic medication for the surrogate marker “lowering blood pressure”). We assessed whether the patients mentioned the corresponding benefit of their medication, whether they mentioned it actively, and whether the mentioned benefit motivated the patients to take their medication as prescribed. We then performed a chi-square test with these data.

Mann–Whitney U tests were performed to assess differences in the PHQ-4 between patients with regard to their knowledge of risks and benefits. The threshold for statistical significance was set at *p* < 0.05.

For each benefit and risk mentioned, patients were asked where they got this information from. Multiple answers were possible. The analysis was carried out at the patient level. Once a patient mentioned a source of information, this was considered a “source of information used.” No further weighing was carried out, taking into account the frequency of the mentions within an individual patient interview.

The confidence interval of 95% was determined for each value.

## 3. Results

### 3.1. Participants

**Figure 1:**
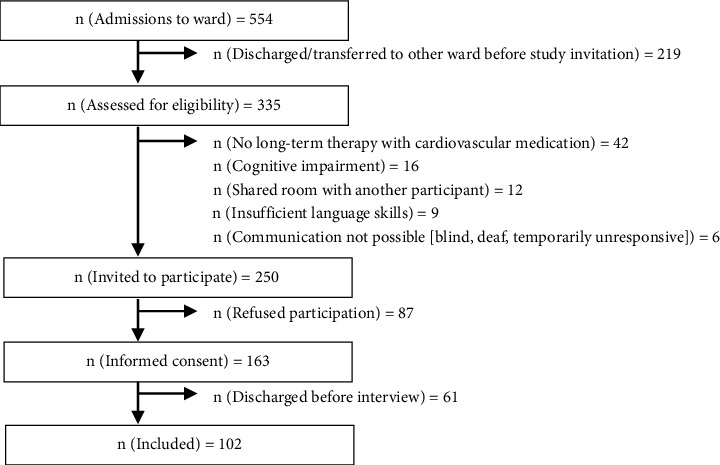
Flow chart of the participating patients.

Of 554 patients admitted to the department of cardiology during the study period, 102 (18%) participated in the interview, and the response rate to an invitation to participate was 41%; a further 24% agreed to participate but could not be interviewed due to organizational problems (Figure 1).  Table 1 shows the demographic data of the participants.

The five most common diagnoses of the study population were “hypertension” (79 patients with diagnosis; 77%), “coronary artery disease/atherosclerosis” (48; 47%), “diabetes mellitus type II/type I/impaired glucose tolerance” (42; 41%), “atrial fibrillation/flutter” (41; 40%), and “Heart failure” (32; 31%). For further information, see Supplementary Appendix 2.

With regard to the drugs defined as “cardiovascular” according to the ATC group, it was found that 12% of the total 548 prescribed cardiovascular drugs belonged to a group of “drugs used in diabetes” (A10). Furthermore, 21% belonged to “antithrombotic agents” (B01), 1% to “antihypertensives” (C02), 15% to “diuretics” (C03), 15% to “beta blocking agents” (C07), 5% to “calcium channel blockers” (C08), 17% to “agents acting on the renin-angiotensin system” (C09), and 13% to “lipid modifying agents” (C10). For details, see Supplementary Appendix 3.

#### 3.1.1. General Benefit-Risk Estimation

41 (40%) patients rated the benefits of their medication as “clearly predominating,” 26 (25%) as “rather predominating.” 5 (5%) patients rated the risks as “rather predominating,” and none as “clearly predominating.” 11 (11%) patients rated their benefits and risks as “balanced.” No specified answers were given by 19 (19%) patients to this question.

#### 3.1.2. Patients' Views on Drug Benefits

**Figure 2:**
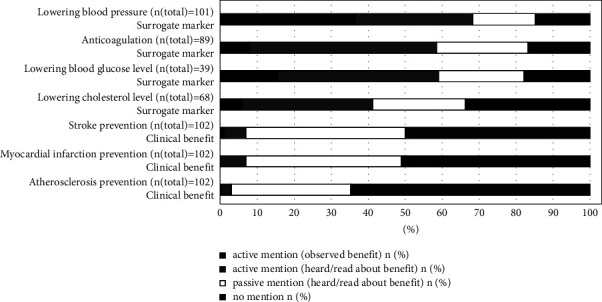
Patients' association of predefined benefits with their therapy. *n* (total) refers to the respective patients with the corresponding cardiovascular medication. Patients mentioned benefits actively without any suggestion or subsequently passively based on a list of suggestions. Patients mentioned surrogate markers significantly more frequently than clinical benefits (actively and passively mentioned: *p* < 0.001; only actively mentioned: *p* < 0.001).

**Figure 3:**
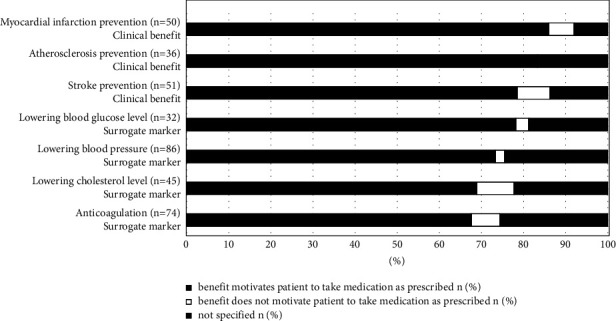
Motivation of associated benefits to take medication as prescribed. *n* (total) refers to the respective patients who mentioned the corresponding benefit. Surrogate markers and clinical benefits did not differ significantly in their patients' motivation for adherence (*p*=0.781).

101 (99%) of the patients mentioned at least 1 drug benefit. In total, the patients mentioned 637 benefits within the median (Q25/Q75) 6 (4/8) per patient. Patients associated 354 metioned benefits (56%) with a particular cardiovascular drug. As shown in Figure 2, the most frequently named benefit was “lowering blood pressure” (87% of the patients with antihypertensive medication). Patients more frequently named surrogate markers than clinical benefits (*p* < 0.001, Figure 2). 460 (72%) of 637 mentioned benefits motivated the patients to take their medication as prescribed. The benefits motivated the patients to take their medication with a range of 68% (anticoagulation) to 86% (myocardial infarction prevention) of the respective patients. We found no difference in motivation between surrogate markers and clinical benefits (Figure 3, *p* = 0.781). The benefit of “lowering blood pressure” was also mentioned as the most frequently observed cardiovascular benefit by 37% of the patients with corresponding medication (Figure 2). Patients most frequently associated the cardiovascular benefit “anticoagulation” with the respective drug (76% of the patients with anticoagulation medication). Additionally, to the predefined benefits, which are shown in Figures 2 and 3, patients mentioned further cardiovascular benefits: diuresis (28 patients; 19 motivated), supporting the cardiovascular system (21; 18), heart rate regulation (8; 6), protection from consequences of atrial fibrillation (4; 4), improvement of cardiac arrhythmia (3; 3), improvement of heart failure (2; 0); and symptom control in angina pectoris (2; 1).

#### 3.1.3. Patients' Views on Drug Risks

**Figure 4:**
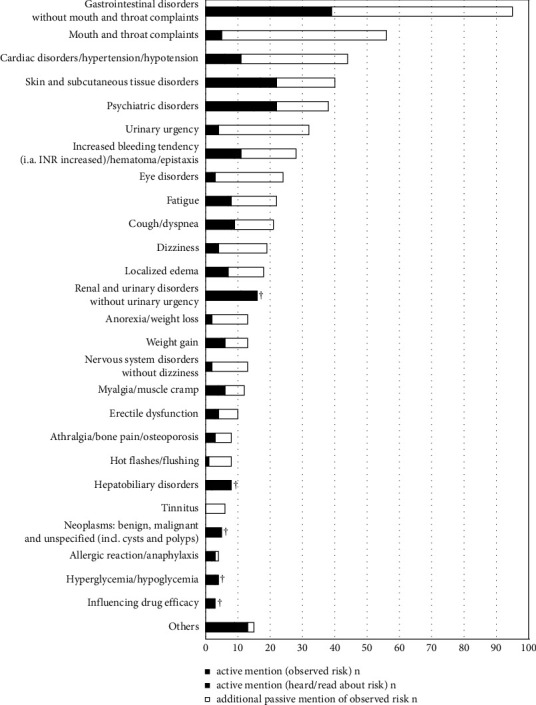
Patients' association of risks with their therapy. Patients mentioned risks actively without any suggestion or passively based on a list of suggestions. Every single mentioned risk was counted and categorized. Multiple risks per patient were therefore possible. Different risks could be categorized into the same AE category per patient. ^†^AE category was not on the predefined list; therefore, passive mention was not possible.

91 (89%) of the patients reported at least 1 risk that they either had experienced or were aware of. In total (actively and passively), the patients reported 575 risks within the median (Q25/Q75) 4 risks (1/8) per patient. 143 (25%) of these risks were associated with a specific cardiovascular drug by the patients. The five most frequently named risk categories were “gastrointestinal disorders” (95 risks with 54 patients affected), “mouth and throat complaints” (56 in 32 patients), “cardiac disorders/hypertension/hypotension” (44 in 29 patients), “skin and subcutaneous tissue disorders” (40 in 32 patients), and “psychiatric disorders” (38 in 23). The risk category most frequently associated with a particular drug was “increased bleeding tendency/hematoma/epistaxis” (86%). 56% of the mentioned risks were perceived as bothersome, 35% as concerning, and 7% caused the patients to take their medication differently than prescribed. Out of the 10 most frequently reported risks, “psychiatric disorders” (particularly addiction, sleep disorders, and depression) were the only risk category that was perceived as concerning in more than 50% (Figure 4; Table 2). Patients who perceived risks as bothersome or concerning had higher PHQ-4 scores than patients who did not (*p*=0.016; Table 3).

#### 3.1.4. Information Sources

The interviewed patients most commonly received information about drug benefits (94 patients, 92%) and risks (46, 45%) from their physician (Table 4). The second most common information source among healthcare professionals was the pharmaceutical staff (27% vs. 14% of the patients). The patients used laymen/media and the package leaflet frequently as sources of information on the benefits of drugs (39% vs. 26%). 42% of the interviewed patients wished for more information about the benefits of their medication and 27% about the risks (Table 4).

## 4. Discussion

Our study analyzed patients' views on the drug benefits and risks of cardiovascular therapy. We performed this study to gain information on how to better support patients with tailored information in their challenge to adhere to their cardiovascular therapy. In this regard, we found that clinical benefits were less frequently mentioned compared to surrogate markers. Both types of benefits motivate patients to take their medication as prescribed. In contrast to this, risks are commonly bothersome and concerning. This was particularly the case in patients with higher PHQ-4 scores. Patients most frequently used their physicians as an information source, particularly for information about the benefits of drugs. They expected to be better informed about the benefits of their therapy in particular. A stronger focus on clinical benefits should be considered by all healthcare professionals (i.e., physicians, but also pharmaceutical staff and nurses).

We decided not to tape-record the interviews in our study. The prompt and thus nearly verbatim documentation of patients´ responses were sufficient for the quantitative purposes of the study we aimed at. Condensation on relevant aspects of the evaluation was easily achieved. On the contrary, an anticipated tape recording could have decreased patients' willingness to participate or, in case of participation, could have led to bias and few free responses. This would have had a counterproductive effect on the results.

### 4.1. Talking More about Drug Benefits

Cardiovascular patients tend to recognize the benefits of their drug therapy rather than the risks. Nevertheless, a small proportion of these patients (16%) do not perceive any difference or even state that risks are predominating. Such concern about risks is known to be a reason for the refusal and discontinuation of pharmacotherapy [27]. Especially with the latter patients, healthcare professionals should be encouraged to talk more about the benefits with their patients. Our findings underline the information gap, i.e., that patients are frequently not well informed about the therapeutic aims of their therapy [28]. Analyzing it more closely, we found that patients are more aware of surrogate markers, e.g., lowering blood pressure, than clinical benefits, e.g., stroke prevention. One explanation approach is that surrogate markers can be observed comparatively easily by the patients themselves. In contrast, the effects of clinical benefits might occur only in the distant future. Patients do not perceive specific symptoms immediately, which can complicate adherence to therapy [29]. Another explanation for the different awareness of surrogate and clinical benefits is that surrogate markers help to distinguish between the different drugs of the combined preventive therapy. As a consequence, they are more often addressed in information and counseling. Patients may consider the surrogate marker to be the main benefit because they are not aware of their lack of knowledge. These findings suggest that healthcare professionals should be encouraged to better emphasize the clinical benefits. This could increase motivation for the patients taking their medication.

### 4.2. Need for Tailored Drug Risk Information

Patient information and counseling about drug risks are justified by ethical and statutory provisions but are also a prerequisite for shared decision-making [30]. More than half of the mentioned drug risks were perceived as bothersome, with one-third as concerning. These alarmingly high rates of negative attributions of drug risks translate to a need for action. Therefore, our results support carefully individualized risk information, which is a challenge for health professionals. In two noticeable examples, we want to emphasize the identification of vulnerable patients or risks for particular diligent counseling and the patient's empowerment in dealing with a risk. First, patients with higher PHQ-4 scores, which go along with a higher tendency for anxiety and depression, were more likely to report risks as bothersome or concerning. In addition, the risk of “psychiatric disorders” was most frequently perceived as concerning. We did not find differences in information demand in patients with different PHQ-4 scores. Nevertheless, our results support risk information strategies that identify vulnerable patients and adapt the information to meet individual requirements. The PHQ-4 score seems to be a valuable short form to identify particular psychiatric vulnerabilities [31].

Second, “an increased tendency to bleed” was also one of the ten most frequently mentioned risks. Conspicuously, patients were hardly concerned about this risk, and in most cases, they were able to assign this risk to a specific drug. This can be explained by the particular complex counseling procedures for anticoagulants because of their status as high-risk drugs [28, 30, 32, 33]. It can be assumed that healthcare professionals intensively discuss bleeding risks and their management with their patients. The patients might then feel more competent in dealing with the risk. Thus, the risks of anticoagulants may be an example of information that is already balanced. It should be considered which strategies could be transferred to other drugs as well.

### 4.3. Patient Information and Counseling in the Context of Laymen Information and Package Leaflets

Digitalization facilitates patient access to a wide range of different information sources. Unfortunately, it is often difficult to distinguish between professional and laymen information in the healthcare sector [34, 35]. Laymen information has shown to incite patient concerns about risks [7, 8]. Such concern can decrease adherence, as shown for statins [9, 10, 36]. It can be assumed that laymen media information more often focuses on risks and thereby contributes to the imbalance between risks and benefits estimation [8, 37]. In this context, it is a reassuring result that the Internet is an information source for only one-fifth of patients, although we expect an increase in the future. Package leaflets are another easy-access information source. Thus, they were used more often by our respondents, especially in cases of risk. Therefore, it is not surprising that package leaflets can increase patients' concerns [23, 38]. They are not the easiest source of information for the patient to understand. Even healthcare professionals misinterpret information on risk frequency and causality in package leaflets [39]. Both information sources appeared unsatisfactory to the patients. Consequently, it can be expected that patients in general seem to appreciate more information [40].

Besides, certain patient characteristics could indicate a further need for intensified or tailored information with respect to the individual patient [11, 12]. We can further conclude that patients should not be left alone with drug information obtained from the Internet or package leaflets. Healthcare professionals should be sensitized and acknowledge their role as a professional information source who can help to prevent misconceptions in their patients. This applies not only to physicians but also to nursing staff and pharmacists who are involved in patient care.

### 4.4. Limitations

Our results are restricted to inpatients of a single hospital who probably had rather good access to healthcare professionals. We evaluated the patients' self-assessed motivation to take their medication as prescribed and whether risks were perceived as bothersome or concerning or causing the patient to take medication differently from the prescription. We thereby did not differentiate by the respective extent. We can only conclude motivation to adhere but not the actual, objective adherence of the patient, which was not the focus of our study. We did not analyze the causality of the mentioned risks. The lists with predefined suggestions of benefits and risks were tailored to cardiovascular drugs and covered frequent or important benefits or risks. Nevertheless, it could not cover all possible benefits and risks. Interpreting the results is further limited because the final interview was not validated; however, it was pretested in advance. Furthermore, it must be noted that due to the semistructured form of the interview, slight variations in the order of questions were accepted.

## 5. Conclusions

Cardiovascular patients rated the expected benefits of their drugs—in most cases based on known surrogate markers—higher than potential risks. Knowledge of benefits frequently motivates patients to take drugs as prescribed. In total, they are more interested in further information on benefits than on risks. Nevertheless, the provided insight into patients' views emphasizes the need for additional information on clinical benefits rather than on surrogate markers and, to a lesser extent, also on drug risks. More precisely, our findings firstly indicate that especially clinical benefit information is not yet fully utilized by healthcare professionals as a valuable resource to motivate the patient to take their medication. They also suggest that the higher PHQ-4 score in patients who perceive drug risks as bothersome or concerning should be considered in order to individualize information and counseling about risks.

## Figures and Tables

**Table 1:** Patient characteristics.

Characteristics	*N*
Age	
Age in years (median (Q25/Q75))	67.5 (56/76)
Min/Max	20/91
Cardiovascular medication	
Cardiovascular drugs (median (Q25/Q75))	5 (4/7)
Min/Max	1/13
Way of patient admission	
Elective (*n* (%))	51 (50%)
Nonelective (*n* (%))	51 (50%)
Gender	
Female (*n* (%))	36 (35%)
Male (*n* (%))	66 (65%)
Highest level of education	
Without graduation (*n* (%))	3 (3%)
Certificate of Secondary Education (*n* (%))	26 (25%)
General Certificate of Secondary Education (*n* (%))	45 (44%)
General qualification for university entrance (*n* (%))	27 (26%)
Not specified (*n* (%))	1 (1%)
Highest level of professional qualification	
Without qualification (*n* (%))	3 (3%)
Vocational training (*n* (%))	74 (73%)
University degree (*n* (%))	19 (19%)
Ph.D. degree (*n* (%))	5 (5%)
Not specified (*n* (%))	1 (1%)
Net income per month	
< 1000 euros (*n* (%))	18 (18%)
1001–2000 euros (*n* (%))	33 (32%)
2001–3000 euros (*n* (%))	22 (22%)
3001–4000 euros (*n* (%))	8 (8%)
4001–5000 euros (*n* (%))	4 (4%)
> 5000 euros (*n* (%))	4 (4%)
Not specified (*n* (%))	13 (13%)
PHQ-4 score^*∗*^ (patient health questionnaire for anxiety and depression)	
0–2 (normal) (*n* (%))	49 (48%)
3–5 (mild) (*n* (%))	23 (23%)
6–8 (moderate) (*n* (%))	19 (19%)
9–12 (severe) (*n* (%))	2 (2%)
Not specified (*n* (%))	9 (9%)

**Table 2:** Frequencies of whether a mentioned risk was perceived as bothersome, concerning, or led the patients to take their medication differently than prescribed. Percentages are based on the total number of mentions of the respective risk.

Reported risk	Total mentions of risk *n*	Risk perceived as bothersome *n* (*n *(%))	Risk perceived as concerning *n* (*n *(%))	Risk resulting in taking medication differently than prescribed *n* (*n *(%))
Gastrointestinal disorders without mouth and throat complaints	95	54 (57%)	30 (32%)	12 (13%)
Mouth and throat complaints	56	33 (59%)	13 (23%)	1 (2%)
Cardiac disorders/hypertension/hypotension	44	20 (45%)	19 (43%)	2 (5%)
Skin and subcutaneous tissue disorders	40	20 (50%)	12 (30%)	2 (5%)
Psychiatric disorders	38	20 (53%)	21 (55%)	2 (5%)
Urinary urgency	32	18 (56%)	7 (22%)	10 (31%)
Increased bleeding tendency (i.a. INR increased)/hematoma/epistaxis	28	12 (43%)	7 (25%)	2 (7%)
Eye disorders	24	14 (58%)	10 (42%)	1 (4%)
Fatigue	22	15 (68%)	7 (32%)	0 (0%)
Cough/dyspnea	21	18 (86%)	10 (48%)	2 (10%)
Dizziness	19	12 (63%)	6 (32%)	1 (5%)
Localized edema	18	13 (72%)	9 (50%)	1 (6%)
Renal and urinary disorders without urinary urgency	16	5 (31%)	10 (63%)	1 (6%)
Anorexia/weight loss	13	4 (31%)	3 (23%)	0 (0%)
Weight gain	13	12 (92%)	4 (31%)	0 (0%)
Nervous system disorders without dizziness	13	9 (69%)	4 (31%)	0 (0%)
Myalgia/muscle cramp	12	9 (75%)	5 (42%)	2 (17%)
Erectile dysfunction	10	5 (50%)	2 (20%)	0 (0%)
Arthralgia/bone pain/osteoporosis	8	4 (50%)	4 (50%)	0 (0%)
Hot flashes/flushing	8	8 (100%)	2 (25%)	0 (0%)
Hepatobiliary disorders	8	0 (0%)	6 (75%)	0 (0%)
Tinnitus	6	4 (67%)	3 (50%)	0 (0%)
Neoplasms: benign, malignant, and unspecified (incl. cysts and polyps)	5	0 (0%)	2 (40%)	0 (0%)
Allergic reaction/anaphylaxis	4	2 (50%)	3 (75%)	0 (0%)
Hyperglycemia/hypoglycaemia	4	1 (25%)	0 (0%)	0 (0%)
Influencing drug efficacy	3	1 (33%)	1 (33%)	1 (33%)
Others	15	7 (47%)	4 (27%)	1 (7%)

**Table 3:** Differences in the PHQ-4-score in patients depending on their answers in the interview.

Questions	PHQ-4 score (median (Q25/Q75)) in patients who answered the questions affirmatively	PHQ-4 score (median (Q25/Q75)) in patients who answered the questions negatively	*p* value^†^
Are you worried about (the respective risk)?/Does (the respective risk) bother you?^††^	3 (1/6) *n*_total_ = 63	1.5 (1/3) *n*_total_ = 22	0.016^*∗*^
Are you motivated by (the respective benefits) to take a drug as prescribed?	2 (1/5) *n*_total_ = 78	PHQ-4 = 2; 6; 6 *n*_total_ = 3^†††^	n.a.
Would you like to get more information on drug risks?	3 (1/6) *n*_total_ = 26	2 (1/4) *n*_total_ = 65	0.160
Would you like to get more information on drug benefits?	3 (1/6) *n*_total_ = 41	2 (1/4) *n*_total_ = 51	0.586

^†^Determined by Mann–Whitney U test. ^††^Due to the limited number of patients who took their medication other than prescribed because of a certain risk, a statistical analysis was not conducted. ^†††^Not appropriate for statistical evaluation.

**Table 4:** Sources of information: number of patients who use and request certain sources of information about benefits and risks concerning their drug therapy (*n*_total_ = 102 patients). Multiple categories were possible.

	Patients' answers regarding drug benefits *n* (*n *(%); 95%–CI)	Patients' answers regarding drug risks *n* (*n *(%); 95%–CI)
*Where did you get the information from?*		
Healthcare professionals	**95 (93%; 88–98%)**	**48 (47%; 37–57%)**
Physician	94 (92%; 87–97%)	46 (45%; 35–55%)
Pharmaceutical staff	28 (27%; 18–36%)	14 (14%; 7–21%)
Nurses	9 (9%; 3–15%)	3 (3%; 0–6%)
Patient information leaflet (PIL)	**27 (26%; 17–35%)**	**37 (36%; 27–45%)**
Laymen (-media)	**40 (39%; 30–48%)**	**41 (40%; 30–50%)**
Relatives/friends/acquaintances	15 (15%; 8–22%)	30 (29%; 20–38%)
Internet	19 (19%; 11–27%)	14 (14%; 7–21%)
Magazines	10 (10%; 4–16%)	12 (12%; 6–18%)
Television	5 (5%; 1–9%)	9 (10%; 3–15%)
*Would you like to get more information about your medicines?*		
Yes (total)	**43 (42%;32–52%)**	**28 (27%; 18–36%)**
No (total)	**56 (55%;45–65%)**	**72 (71%; 62–80%)**
Not specified	2 (2%; 0–5%)	1 (1%; 0–3%)
No matter	1 (1%; 0–3%)	1 (1%; 0–3%)
*Who would you like to be informed by?*		
Physician	40 (39%; 30–48%)	25 (25%; 17–33%)
Pharmaceutical staff	35 (34%; 25–43%)	19 (19%; 11–26%)
Nurses	19 (19%; 11–27%)	14 (14%; 7–21%)
Others	6 (6%; 1–11%)	9 (9%; 3–15%)

## Data Availability

Due to ethical concerns regarding patient privacy, supporting data cannot be made available.

## Supplementary Materials

Supplementary Materials
*Appendix 1.* Semistructured questionnaire. Original questions from the different interview parts are presented. *Appendix 2.* Diagnoses of the study population related to the cardiovascular setting. *Appendix 3*. Cardiovascular drugs and the frequency of their prescription in the study population.
