# Supplementary material for: Assessment of Patients' Views on Drug Benefits and Risks: An Interview Study with Cardiovascular Patients
Source: Int J Clin Pract. 2022 Nov 14;2022:6585271. doi: 10.1155/2022/6585271 (PMC9678459; doi:10.1155/2022/6585271)
Supplement: Supplementary Materials — Appendix 1. Semistructured questionnaire. Original questions from the different interview parts are presented. Appendix 2. Diagnoses of the study population related to the cardiovascular setting. Appendix 3. Cardiovascular drugs and the frequency of their prescription in the study population. [file 6585271.f1.doc]

**Supplementary Material**

**Appendix 1:** Semi-structured questionnaire. Original questions of the different interview parts are presented.

|  | **Topics** | **Questions** | |
| --- | --- | --- | --- |
| **Introduction Part** | | | |
|  |  | Patient is asked for:  • Current medication | |
| **Semi-structured Part** | | | |
| (i) | General estimation of therapeutic benefits and risks | • How would you compare benefits and risks of your medication? | |
| (ii) | Association of therapeutic benefits of applied drugs and their self-evaluated influence on therapy adherence | • Have you ever **observed** benefits of your drugs? ***active report from the patient*** | |
|  |  |  | • What effects did you observe/were observed?  • Have you ever heard or read about any of the benefits observed before?  • If yes: Where did you get this information from?  • Let’s focus on these observed benefits. Do you associate them with certain drugs?  • If yes: With which ones?  • Are you motivated by all or only certain positive experiences to take a drug as prescribed? |
|  |  | • Have you (in addition to your own experiences) **heard or read** about the benefits of you drugs? ***active report from the patient*** | |
|  |  |  | *Following questions as above.* |
|  |  | • Have you (also) ever heard any of the benefits listed here in connection with your drugs? *(presentation of list with possible benefits of cardiovascular medication)* ***passive report from the patient*** | |
|  |  |  | *Following questions as above.* |
| (iii) | Association of the risks of applied drugs and their self-evaluated influence on therapy adherence | • Have you ever **observed** adverse events caused by your drugs? ***active report from the patient*** | |
|  |  |  | • What did you observe/was observed?  • Have you ever heard or read about any of the side effects observed before?  • If yes: Where did you get this information from?  • Let’s focus on these observed adverse events. Do you associate them with certain drugs?  • If yes: With which ones?  • Does this lead or did it lead to you taking your drugs another way than prescribed by your doctor?  • Are you worried about this?  • Does it bother you? |
|  |  | • Have you ever **heard or read** anything else about adverse events that your drugs might have? ***active report from the patient*** | |
|  |  |  | *Following questions as above without “Does it bother you?”* |
|  |  | • This is a list of various adverse events. Have you ever **observed** any of them caused by your drugs? *(presentation of list of possible adverse events caused by cardiovascular medication)* ***passive report from the patient*** | |
|  |  |  | *Following questions as above.* |
| (iv) | Used and requested information sources *(Used information sources were also asked for in parts (iii) and (iv).)* | • Would you like to get more information about the adverse events of your drugs? | |
|  |  |  | • Who would you like to be informed by? |
|  |  | • Would you like to get more information about the benefits of your medicines? | |
|  |  |  | • Who would you like to be informed by? |
| **Completion Part** | | | |
|  |  | Patient is asked for:  • Sociodemographic data  • Completion of PHQ-4 questionnaire (in written form) | |

**Appendix 2. Diagnoses of the study population related to cardiovascular setting**

| **Diagnoses related to cardiovascular setting*** | **Number of patients with diagnosis [n]** | **[n(%)]**  ***n_total_=102*** |
| --- | --- | --- |

| Hypertension | 79 | 77,5% |
| --- | --- | --- |
| Coronary artery disease/atherosclerosis | 48 | 47,1% |
| Diabetes mellitus type II/type I/impaired glucose tolerance | 42 | 41,2% |
| Atrial fibrillation/flutter | 41 | 40,2% |
| Heart failure | 32 | 31,4% |
| Cardiomyopathy | 29 | 28,4% |
| Dyslipidemia | 28 | 27,5% |
| Heart valve failure | 24 | 23,5% |
| Myocardial infarction | 23 | 22,5% |
| Angina pectoris | 20 | 19,6% |
| Cardiac arrhythmia (without atrial fibrillation/flutter) | 18 | 17,6% |
| Obesity | 18 | 17,6% |
| Surgical interventions on the heart | 14 | 13,7% |
| Stroke/TIA | 12 | 11,8% |
| AV block/left bundle branch block/bifascicular block | 10 | 9,8% |
| Peripheral artery disease (PAD) | 10 | 9,8% |
| Pulmonary hypertension | 9 | 8,8% |
| Depression | 8 | 7,8% |
| Chronic venous insufficiency/varicosis | 6 | 5,9% |
| Coronary heart disease (CHD) | 6 | 5,9% |
| Cardiac arrest/cardiogenis shock | 6 | 5,9% |
| Pulmonary embolism | 5 | 4,9% |
| Persitent foramen ovale | 4 | 3,9% |
| Venous thrombosis | 4 | 3,9% |
| Syncope/orthostatic dysregulation | 4 | 3,9% |
| Other  (In-stent restenosis, Pacemaker pocket infection, Peromyocarditis, Aortic ectasia, Supply of LifeVest wearable defibrillator, Arteriovenous fistula, Transfusion-requiring bleeding anemia caused by phenprocoumon overdose, Psychocardiological presentation in case of recurrent painful paresthesia of unknown origin, Atrial septal aneurysm, Post atrial septal defect) | 10 | 9,8% |

*Including depression due to the methodological approach of the study, which incorporates the assessment of the PHQ4-score.

**Appendix 3. Cardiovascular drugs and the frequency of their prescription in the study population**

| **Cardiovascular Drugs** | | **ATC-Code** | **Number of prescriptions [n]** | **[n (%)*]** |
| --- | --- | --- | --- | --- |
| **Drugs used in diabetes** | | **A10** | **68** | **12,4%** |
|  | Metformin | A10BA02 | 15 | 2,7% |
|  | Insulin glargine | A10AE04 | 11 | 2,0% |
|  | Insulin glulisine | A10AB06 | 9 | 1,6% |
|  | Sitagliptin | A10BH01 | 8 | 1,5% |
|  | Insulin (human; fast-acting) | A10AB01 | 5 | 0,9% |
|  | Empagliflozin | A10BK03 | 4 | 0,7% |
|  | Insulin detemir | A10AE05 | 3 | 0,5% |
|  | Insulin aspart | A10AB05 | 3 | 0,5% |
|  | Glimepride | A10BB12 | 2 | 0,4% |
|  | Dulaglutide | A10BJ05 | 2 | 0,4% |
|  | Insulin lispro | A10AB04 | 1 | 0,2% |
|  | Liraglutide | A10BJ02 | 1 | 0,2% |
|  | Dapagliflozin | A10BK01 | 1 | 0,2% |
|  | Insulin (human; long-acting) | A10AE01 | 1 | 0,2% |
|  | Metformin and sitagliptin | A10BD07 | 1 | 0,2% |
|  | Repaglinide | A10BX02 | 1 | 0,2% |
| **Antithrombotic agents** | | **B01** | **117** | **21,4%** |
|  | Acetylsalicylic acid | B01AC06 | 49 | 8,9% |
|  | Apixaban | B01AF02 | 20 | 3,6% |
|  | Clopidogrel | B01AC04 | 12 | 2,2% |
|  | Phenpropcoumon | B01AA04 | 11 | 2,0% |
|  | Edoxaban | B01AF03 | 7 | 1,3% |
|  | Ticagrelor | B01AC24 | 7 | 1,3% |
|  | Rivaroxaban | B01AF01 | 5 | 0,9% |
|  | Tinzaparin | B01AB10 | 2 | 0,4% |
|  | Dabigatran etexilate | B01AE07 | 1 | 0,2% |
|  | Certoparin | B01AB13 | 1 | 0,2% |
|  | Prasugrel | B01AC22 | 1 | 0,2% |
|  | Enoxaparin | B01AB05 | 1 | 0,2% |
| **Antihypertensives** | | **C02** | **7** | **1,3%** |
|  | Moxonidine | C02AC05 | 5 | 0,9% |
|  | Doxazosin | C02CA04 | 1 | 0,2% |
|  | Bosentan | C02KX01 | 1 | 0,2% |
| **Diuretics** | | **C03** | **84** | **15,3%** |
|  | Torasemide | C03CA04 | 46 | 8,4% |
|  | Spironolacton | C03DA01 | 16 | 2,9% |
|  | Hydrochlorothiazide | C03AA03 | 11 | 2,0% |
|  | Eplerenone | C03DA04 | 4 | 0,7% |
|  | Xipamide | C03BA10 | 4 | 0,7% |
|  | Furosemide | C03CA01 | 1 | 0,2% |
|  | Bendroflumethazid and amilorid | C03EA33 | 1 | 0,2% |
|  | Bemetizid and triamteren | C03EA16 | 1 | 0,2% |
| **Beta blocking agents** | | **C07** | **83** | **15,1%** |
|  | Metoprolol | C07AB02 | 38 | 6,9% |
|  | Bisoprolol | C07AB07 | 30 | 5,5% |
|  | Carvedilol | C07AG02 | 9 | 1,6% |
|  | Propranolol | C07AA05 | 2 | 0,4% |
|  | Metoprolol and ivabradine | C07FX05 | 2 | 0,4% |
|  | Nebivolol | C07AB12 | 2 | 0,4% |
| **Calcium channel blockers** | | **C08** | **25** | **4,6%** |
|  | Amlodipine | C08CA01 | 16 | 2,9% |
|  | Lercanidipine | C08CA13 | 5 | 0,9% |
|  | Verapamil | C08DA01 | 2 | 0,4% |
|  | Felodipine | C08CA02 | 1 | 0,2% |
|  | Nitrendipine | C08CA08 | 1 | 0,2% |
| **Agents acting on the renin-angiotensin system** | | **C09** | **93** | **17,0%** |
|  | Ramipril | C09AA05 | 31 | 5,7% |
|  | Candesartan | C09CA06 | 17 | 3,1% |
|  | Valsartan | C09CA03 | 17 | 3,1% |
|  | Valsartan and sacubitril | C09DX04 | 7 | 1,3% |
|  | Enalapril | C09AA02 | 4 | 0,7% |
|  | Ramipril and amlodipine | C09BB07 | 3 | 0,5% |
|  | Olmesatan medoxomil, amlodipine and hydrochlorothiazide | C09DX03 | 2 | 0,4% |
|  | Valsartan and amlodipine | C09DB01 | 2 | 0,4% |
|  | Lisinopril | C09AA03 | 2 | 0,4% |
|  | Valsartan, amlodipine and hydrochlorothiazide | C09DX01 | 2 | 0,4% |
|  | Losartan | C09CA01 | 1 | 0,2% |
|  | Irbesartan | C09CA04 | 1 | 0,2% |
|  | Valsartan and hydrochlorothiazide | C09DA23 | 1 | 0,2% |
|  | Ramipirl and hydrochlorothiazide | C09BA25 | 1 | 0,2% |
|  | Candesartan and hydrochlorothiazide | C09DA26 | 1 | 0,2% |
|  | Irbesartan and hydrochlorothiazide | C09DA24 | 1 | 0,2% |
| **Lipid modifying agents** | | **C10** | **71** | **13,0%** |
|  | Simvastatin | C10AA01 | 29 | 5,3% |
|  | Atorvastatin | C10AA05 | 26 | 4,7% |
|  | Pravastatin | C10AA03 | 6 | 1,1% |
|  | Ezetimibe | C10AX09 | 4 | 0,7% |
|  | Simvastatin and ezetimibe | C10BA02 | 3 | 0,5% |
|  | Atorvastatin and ezetimibe | C10BA05 | 1 | 0,2% |
|  | Fluvastatin | C10AA04 | 1 | 0,2% |
|  | Lovastatin | C10AA02 | 1 | 0,2% |

*Percentage in relation to total number of drug prescriptions defined as "cardiovascular medication" (n=548 (56.1% of total prescriptions(n=976)).
